# Neglected Branches of Medical Study

**Published:** 1875-11

**Authors:** 


### Neglected Branches of Medical Study.

When we scan the schedules of lectures in the medical colleges in their busiest seasons, we find an amount of occupation outlined for the whole didactic course, apparently over-sufficient for the grasp of ordinary scholastic minds. If we had not ourselves passed through the same ordeal, we might naturally wonder what time was left for the student, wearied with a continuous train of lectures, clinics, dissections and examinations, to such slight personal attentions as are embraced in the provinces of eating, drinking and sleeping. That the branches taught must be considered by their teachers good and amply sufficient, may be inferred from the fact that the best of our schools have plodded along in the same groove for a number of years, with but little change in the programme. So far as length of study, methods of examination of candidates for graduation, and similar very practical matters are concerned, we have at this moment nothing to observe; these have often been discussed, and are still amenable to a just and rigid scrutiny. It might seem like a superfluous criticism for us to intimate that this mass of learning, which is to be packed away in a winter's session, has in it any element of insufficiency; and yet it is evident, at the most superficial glance, that several branches which may in due season become all-important to the practitioner, receive no attention at the hands of the lecturer, or are clothed in the scantiest of material to hide the nakedness of their treatment. Notably among these may be mentioned Medical Jurisprudence, Pharmacy and Practical Hygiene, while Chemistry is too often taught tediously in all its non-medical minutiæ, its medical relations being accidently hinted at only when a convenient occasion arises. The History of Medicine is necessarily pushed aside, far out of sight, by the more practical branches, and left as an after-study for the curious and an accomplishment for the professional virtuso. Half a century ago, however, when the University of Virginia was founded, even this last subject was embraced, with the other neglected branches just named, in the curriculum of instruction. Needing most of these important aids when he settles down to the sterner realities of medical life, the graduate finds when he has most urgent occasion for their service, that some of the tools which he is expected to skillfully handle are rusty from disuse, and that he must hastily get them in order for the emergency in which they are to become of practical utility, after his own untutored fashion, perhaps nervously and imperfectly, at a time when the eyes of a whole community may be fixed upon him. He may be pardoned at such a moment for thinking that the system of instruction which seemed so perfect to him as a student must have been full of glaring deficiencies.

So far as the teaching of Pharmacy is concerned, there is no reason why it should be so thoroughly isolated from the medical courses, and left entirely to the pharmaceutical colleges. Thousands of medical men throughout the country are necessarily their own apothecaries, and many of them travel in a circle as prescribers in the well-trodden footsteps of their routine-loving preceptors, often confining themselves to the employment of a few old-fashioned remedies, merely because they have never had imparted to them during their student-life the general principles of manipulation and preparation of drugs and chemicals. They are only roused to a just appreciation of the claims of Practical Hygiene, when a virulent epidemic, "at one fell swoop," sweeps away their patients and their own reputation as skilful practitioners. They then impatiently overhaul their text-books, and "when found, make a note of it," for the next epidemic, which may never visit them; but their attention has never been excited to the means of prevention of the more common diseases which are daily present at their very elbow. Medical Jurisprudence is shamefully neglected; a mere smattering of information as to its toxicological relations is sometimes acquired in the schools from incidental mention by the teacher of the department of Materia Medica, but the great mass of the profession is wofully ignorant of the medico-legal complications in which practitioners may sometimes be seriously involved. It should be borne in mind that medical experts are not the only ones who are likely to be summoned as witnesses in judicial cases. Every village doctor may some time or other occupy a creditable or a ridiculous position on the witness-stand, according to the extent and variety of his information on questions of medico-legal import. In many of the schools, Medical Jurisprudence is tacked on as a supplement to another branch, in some of them a teacher being in the same session professor of both Institutes of Medicine and Medical Jurisprudence; but, even when so taught, it occupies but a subordinate place, not at all commensurate with its merits, and under such circumstances still deserves to be classed among the almost neglected branches. Occasionally it is offered to slender summer classes as a temporary placebo, but although sometimes excellently taught, it even then does not assume its deserved prominence, and fails to reach in its influences the greater masses of the more important winter classes, from which the ranks of the profession are most numerously recruited.—*Medical Record Editorial.*

